# Molecular Mechanisms and Metabolomics of Natural Polyphenols Interfering with Breast Cancer Metastasis

**DOI:** 10.3390/molecules21121634

**Published:** 2016-12-17

**Authors:** Yingqian Ci, Jinping Qiao, Mei Han

**Affiliations:** Key Laboratory of Radiopharmaceuticals, Ministry of Education, College of Chemistry, Beijing Normal University, Beijing100875, China; ciyingqian1127@163.com

**Keywords:** natural polyphenols, breast cancer metastasis, metabolomics

## Abstract

Metastatic cancers are the main cause of cancer-related death. In breast primary cancer, the five-year survival rate is close to 100%; however, for metastatic breast cancer, that rate drops to a mere 25%, due in part to the paucity of effective therapeutic options for treating metastases. Several in vitro and in vivo studies have indicated that consumption of natural polyphenols significantly reduces the risk of cancer metastasis. Therefore, this review summarizes the research findings involving the molecular mechanisms and metabolomics of natural polyphenols and how they may be blocking breast cancer metastasis. Most natural polyphenols are thought to impair breast cancer metastasis through downregulation of MMPs expression, interference with the VEGF signaling pathway, modulation of EMT regulator, inhibition of NF-κB and mTOR expression, and other related mechanisms. Intake of natural polyphenols has been shown to impact endogenous metabolites and complex biological metabolic pathways in vivo. Breast cancer metastasis is a complicated process in which each step is modulated by a complex network of signaling pathways. We hope that by detailing the reported interactions between breast cancer metastasis and natural polyphenols, more attention will be directed to these promising candidates as effective adjunct therapies against metastatic breast cancer in the clinic.

## 1. Introduction

**Figure 1:**
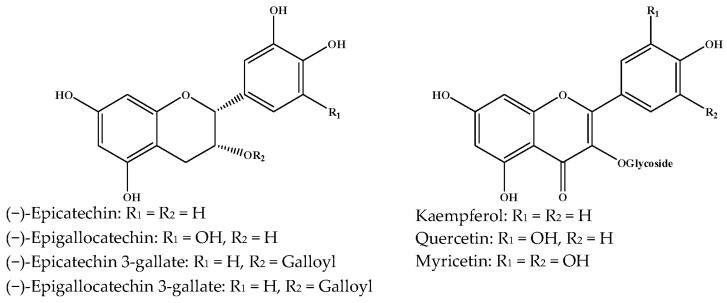
Structures of the major catechins and natural polyphenols.

Catechins, generally known as tea polyphenols and comprising approximately 1/3 of the constituents of green tea), are one of the most attractive natural compounds because of their bioavailability [1]. Natural polyphenols show a very broad spectrum of biological activities, including antioxidant-related activity [2]. Catechins contain characteristic polyphenolic compounds, (−)-epigallocatechin-3-gallate (EGCG), (−)-epigallocatechin (EGC), (−)-epicatechin-3-gallate (ECG), and (−)-epicatechin (EC), shown in Figure 1 [1,3]. Other natural polyphenols such as kaempferol, quercetin, myricetin, and their glycosides (mono-, di-, and tri-) are also present in tea (Figure 1) [4], and tea also includes other natural polyphenols, such as baicalein, resveratrol, curcumin and so on. The analogues of catechins exhibit health benefits that protect against many life-threatening diseases, including cardiovascular diseases, chronic inflammation, and cancer [5], through regulation of various signaling pathways [6]. There is evidence that natural polyphenols can inhibit cancer cell proliferation and metastases, induce apoptosis, and exert chemopreventive activities [7,8].

**Figure 2:**
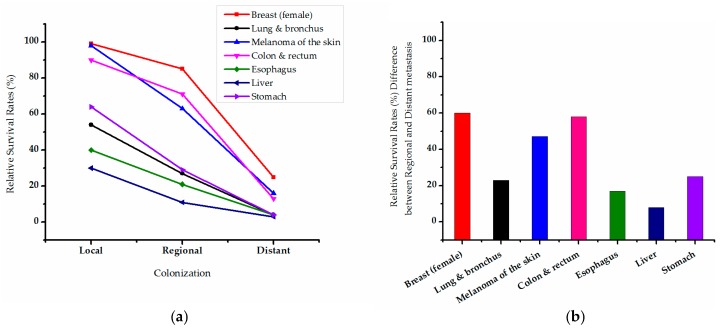
(**a**) Five-year Relative Survival Rates* (%) by Stage at Diagnosis, US, 2004–2010. Local: an invasive malignant tumor confined to primary organ. Regional: a malignant tumor that has extended into surrounding organs or tissues and regional lymph nodes. Distan**t**: a malignant tumor that has spread to parts of the body remote from the primary cancer, or via the lymphatic system to distant lymph nodes; (**b**) The difference in relative survival rates between Regional and Distant metastases, and Value (%) = Regional–Distant. Rates* are adjusted for normal life expectancy and are based on cases diagnosed in the SEER 18 areas from 2004 to 2010, all followed through 2011, and the data are from the American Cancer Society, Inc., Surveillance Research, 2015.

Cancer metastases are the major cause of cancer mortality. Metastatic breast cancer is one of the deadliest types of cancers worldwide in women with a mortality rate greater than 2.1 per million cases annually [9]. We use published cancer statistics in the US in the SEER 18 areas from 2004 to 2010 as an example. Figure 2 shows the five-year relative survival rates of all malignant tumors have significantly improved, especially when focusing on distant metastases (all metastatic cancers had a 5 year survival rate of less than 40%). Although the survival rate of primary breast cancer is now close to 100%, it decreases to only 25% once distant metastasis has occurred (Figure 2a). The presence of metastases causes a sharp drop in survival rate among other cancers as well. Figure 2b shows the difference in five-year relative survival rates between regional and distant metastases (breast cancer and colon and rectum cancer are both approximately 60%). The highest increase in mortality due to distant metastasis occurs in breast cancer. Unfortunately, the only antimetastatic therapeutics in clinical practice, including anthracyclines, taxanes, and trastuzumab, have little efficacy, so it is imperative to find effective drugs for cancer metastasis (data from American Cancer Society, Inc., Surveillance Research, 2015 [10]).

**Figure 3:**
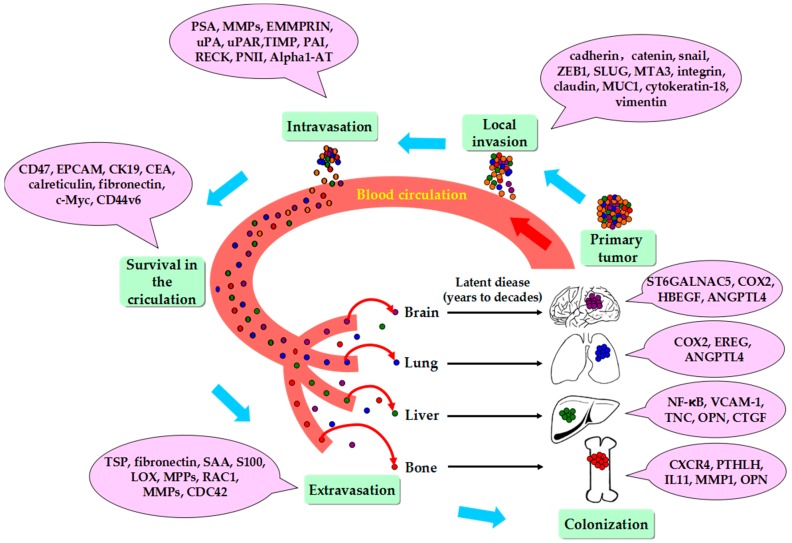
Simplified summarizes of metastatic cascade of breast cancer and genes involving in the process of metastasis. Cancer-driven events in the metastatic cascade are delineated in time and space in a counter-clockwise beginning with the primary cancer, and then intravasation into the blood circulation, active or passive migration toward the target organ, embedding into a capillary bed, attachment to the endothelium, and extravasation, which leads to infiltration into the underlying tissue, and expansion in the target microenvironment. MMPs, matrix metalloproteinases; ZEB1, zinc finger E-box-binding homeobox 1; SLUG, zinc finger protein SNAI2; MTA3, metastasis-associated protein; MUC1: mucin 1, cell surface-associated; PSA, prostate-specific antigen; EMMPRIN: extracellular matrix metalloproteinase inducer; uPA: urokinase plasminogen activator; uPAR: urokinase plasminogen activator receptor; PAI: plasminogen activator inhibitor; RECK: reversion-inducing cysteine-rich protein with kazal motifs; PN-II: protease nexin-II; alpha1-AT: alpha 1-antitrypsin; EPCAM, epithelial cell adhesion molecule; CK19, Cytokeratin 19; CEA, carcinoembryonic antigen; SAA, serum amyloid A; LOX, protein-lysine 6-oxidase; RAC1, Ras-related C3 botulinum toxin substrate1; cdc42, cell division control protein 42 homolog; ST6GALNAC5, AGPTL4: promoting seeding; promoting colonization: OPN, CXCR4; COX-, CXCL12/CXCR4: proinflammatory molecules and chemokines/receptors; VCAM-1, TNC, OPN: adhesion and extracellular matrix molecules; SRC, NF-κB: intracellular signaling proteins. The data adapted from the study of Bos and Zhou [8,14].

Cancer metastasis is a very complicated process. Cancer cells can enter the circulatory system, becoming invasive and motile long before a tumor is found. Only a small proportion of cancer cells manage to permeate regional and distant organs, a fundamental step for eventual relapse. Therefore, a primary cancer might have already seeded regional and distant organs with thousands of cancer cells [11]. Various studies have explored the processes of cancer metastasis in animal models. The metastatic cascade begins with the dissemination of metastasis-competent cells from the primary tumor, intravasation into the blood circulation, active or passive migration toward the target organ, embedding into a capillary bed, attachment to the endothelium, and extravasation, which leads to infiltration into the underlying tissue, and expansion in the target microenvironment (Figure 3) [12]. Generally, for breast cancer metastasis, an invasive tumor often metastasizes to lung, liver, bone, and brain. Metastasis occurs less commonly in pleura, lymph node and spleen [13].

Based on the complicated process of breast cancer metastasis, which involves multiple targets and organs, and the interventional effect of natural polyphenols in vivo, more scientific and advanced technologies, such as metabolomics, should be used to address this delicate process. Metabolomics, involving the comprehensive characterization of metabolism and metabolites in biological systems, is an emerging ‘omics’ science technology. Recent developments in metabolomics technologies are increasingly being utilized to discover molecular mechanisms of disease, customize drug treatments, identify novel drug targets and monitor therapeutic outcomes [15]. In this review, we discuss how natural polyphenols can modulate signaling pathways that are necessary for invasive behavior and metastasis of breast cancer in vitro and in vivo, and then, we briefly summarize the studies of tea catechin on endogenous metabolites using metabolomics technologies.

## 2. The Molecular Mechanism of Natural Polyphenols on Breast Cancer Metastasis In Vitro

Over the past two decades, it has been demonstrated that natural polyphenols are beneficial for both chemoprevention and chemosensitization [16]. Large numbers of studies have concentrated on the ability of natural polyphenols to inhibit cancer cell proliferation and related mechanisms. Increasingly, more and more research has illustrated that natural polyphenols can suppress cancer cell invasion and migration, revealing the therapeutic potential of natural polyphenols against cancer metastases [8]. The tumorigenesis and metastasis need the participation of many molecular pathways that are important in the treatment of every step of cancer progression. Some proteins and enzymes exert effects only on the metastasis ability, and many of these are correlated with metastatic colonization [17].

This review pays special attention to the increasing evidence that natural polyphenols can block migration and invasion of breast cancer cells through a series of molecular mechanisms, including the down-regulation of matrix metalloproteinases (MMPs) expression [18], the regulatory effects of epithelial-to-mesenchymal transition (EMT), the suppression of vascular endothelial growth factor (VEGF) signalling and cancer angiogenesis, the inhibition of nuclear factor-kappa B (NF-κB), and the mammalian targeting function of rapamycin (mTOR), as well as other signalling pathways. The results of those studies are summarized in Table 1.

### 2.1. MMPs

MMPs are a family of the metzincin group of enzymes that share the conserved zinc-binding motif in their catalytic active site [19]. MMPs participate in crucial pathological processes such as cancer, cardiovascular disease and neurological disorders. MMPs play an important role in tissue reconstruction near proliferating cells of malignant neoplasms during cancer metastasis [20]. In particular, MMPs facilitate angiogenesis, cancer cell invasion and metastasis [21]. Large studies have investigated the prognostic value of MMPs in breast cancer metastasis [22,23,24].

#### 2.1.1. MMPs Involving Tissue Inhibitor of Matrix Metalloproteinase

Significantly, low levels of tissue inhibitor of matrix metalloproteinase-3 (TIMP-3) protein expression in breast cancer has been reported to be correlated with an aggressive cancer phenotype [25]. EGCG and Green tea polyphenols (GTP) mediate epigenetic activation of TIMP-3 levels, resulting in suppression of invasiveness and gelatinolytic activity of MMP-2 and MMP-9 in MDA-MB-231 and MCF-7 breast cancer cells [26]. Oleuropein, the main polyphenol in olive oil, has anti-metastatic effects. Studies have found that TIMP-1, -3, and -4 were over-expressed in MDA-cells after incubation with oleuropein (200 μg/mL), while MMP-2 and MMP-9 genes were down-regulated [27]. Piceatannol, a polyphenol that is found in grapes, berries and red wine, exhibits anti-metastatic activities. Similar to EGCG and GTP, the anti-metastatic effect of piceatannol involved increased protein levels of TIMP-2, which decreases MMP-9 activity [28]. Japanese quince fruit juice is used as a food additive, and its polyphenol extract also decreased MMP-9 activity through the stimulation of TIMP-1 expression [29].

#### 2.1.2. MMPs Involving Common Signaling Pathways

Previous studies have shown that AKT is one of the major pathways activated by MMP‑9 expression [74]. The inhibitory effect of EGCG and GTP on the invasion of MDA-MB-231 cells was associated with decreased AKT phosphorylation, and down-regulation of MMP-9 expression [33]. In addition, EGCG and EGC also inhibited heregulin-β1 (HRG)-induced migration and invasion of MCF-7 cells, owing to EGCG down-regulation of ErbB2/ErbB3/PI3K/Akt signaling, EGC exerted these effects through pathways involved in the inhibition of ErbB2/ErbB3 but not Akt [30]. These data suggest that EGCG and EGC reduce MMP‑9 expression through different signalling pathways. Kaempferol can inhibit cancer cell invasion by interrupting the PKCδ/MAPK/AP-1 cascade and subsequently down-regulating MMP-9 expression in MDA-MB-231 human breast carcinoma cells [35]. Baicalein is a natural polyphenols sourced from the root of *Scutellaria baicalensis*, widely used in Chinese herbal medicine. Recent studies have demonstrated that *S. baicalensis* alone, or in combination with other herbs, can inhibit cancer cell growth and induce apoptosis in breast carcinoma cell lines [75]. In MDA-MB-231 cells, treatment with baicalcin down-regulates the expression of MMP-2/9 through the MAPK signalling pathway [38]. Oroxylin A, one of the main bioactive natural polyphenols of *S. radix*., suppresses the invasive abilities of MDA-MB-435 cells by down-regulating the expression of MMP-2 and MMP-9, interfering with phosphorylation of extracellular regulated protein kinases 1/2 (ERK1/2) [37].

Resveratrol, a polyphenol naturally produced by many fruits, reduced growth factor heregulin-β1 (HRG-β1)-mediated MMP-9 expression, phosphorylation of ERK1/2 and invasion in MCF-7 breast cancer cells. These results suggest that the inhibitory effect of resveratrol on MMP-9 expression and invasion is in part associated with down-regulated mitogen-activated protein kinases, MAPK/ERK signaling [41]. Tang and colleagues confirmed that resveratrol significantly inhibited the migration and invasion of MDA-MB-435 ER-negative human breast cancer cells, insulin-like growth factor (IGF-1)-mediated MMP-2 expression, and PI-3K/Akt signaling pathway activation [40,42]. Connective tissue growth factor (CTGF), also known as CCN2, is a member of the CCN family [76]. Down-regulation of CTGF in lung adenocarcinomas [77] and colon cancers [78] is related to invasion and metastasis both in vitro and in vivo. *Nelumbonucifera gaertn*, known as lotus, is used as a medicinal herb in Eastern Asia [79]. Recently, researchers have explored the molecular mechanisms of *N. nucifera* leaf extract on metastasis in breast cancer from the perspective of CTGF. The results demonstrated that down-regulation of CTGF expression in MDA-MB-231 cells markedly attenuated PI3K-AKT-ERK activation, consequently reducing the expression of MMP-2 [64].

#### 2.1.3. MMPs Involving NF-κB

The NF-κB family of transcription factors are key regulators of immune responses, inflammation, and cancer [80]. Sufficient research has demonstrated that NF-κB signaling pathways are closely related to cancer metastasis, suggesting that inhibition of NF-κB activity would disrupt the metastatic potential of mammary epithelial cells in a model system [81]. NF-κB regulates genes linked to cell motility, invasion and metastasis, including the genes encoding MMPs [82]. Curcumin, commonly used polyphenol species, is sourced from the turmeric plant (*Curcuma longa*). Pre-treatment with curcumin specifically blocked TPA-stimulated NF-κB and AP-1 activation, subsequently inhibiting TPA-induced MMP-9 expression in MCF-7 cells [47]. Previous reports have indicated that NF-κB activation can up-regulate MMP-9 [83]. Demethoxycurcumin (DMC) is a natural polyphenolic compound found in curcuma species [84]. Treatment with DMC inhibits expression of MMPs by mediating the DNA binding activity of NF-κB [52]. Dendrosomal curcumin can inhibit MMP-9 expression in breast tumor, the brain, the lung, the liver and the spleen through the suppression of NF-κB in an animal model of metastatic breast cancer [48,50].

Another active ingredient of green tea, **EC**, can down-regulate MMP-9 expression explaining the inhibitory effect of EC on invasion of cancer cells into embryonic stem cell-derived, vascularized tissues [34]. *Murraya koenigii*, a whitish moss, grows in dense cushion with long spongy leaves, in dampy soil or as epophytes. The methanolic extract remarkably inhibited the adhesion, migration and invasion of MCF-7 cells, partially through the inhibition of MMP-2 and MMP-9 activities [65].

### 2.2. Anti-Angiogenesis

Tumor angiogenesis is necessary for the metastatic process because cancer cells leaving primary sites utilize the route provided by newly formed blood vessels [85]. VEGF is a potent angiogenic factor and can be secreted by various types of cancer cells of breast, lung, gastrointestinal tract and others [86]. Previous research has stressed the major role played by VEGF in the proliferation and metastasis of endothelial cells and in neovascularization. Previous studies have shown that natural polyphenols can inhibit cancer metastasis by interfering with the VEGF signalling pathway.

Evening primrose flavanol preparation (EPFP) decreased MDA-MB-231 invasiveness by causing a reduction in VEGF expression at 100 μM gallic acid equivalents. Furthermore, the studies observed a pronounced inhibition of Ki-67 gene expression, the product of which is a universal marker of angiogenesis in breast cancer patients [87]. It was concluded that EPFP treatment reduced the expression levels of Bcl-2, VEGF and 2 transcription factors (c-Jun, c-Fos) via modulation of mRNA expression, which ultimately lead to inhibition of invasiveness by suppressing angiogenesis [69]. Phosphoinositide 3-kinase (PI3K)/Akt signaling is activated and phosphorylated during angiogenesis, which induces the expression of cancer growth related proteins and additional angiogenic factors [88]. Grape skin extract inhibits phosphorylation of Akt, PDK1, p38 and ERk1/2, which partly inhibited cancer angiogenesis and suppressed 4T1 cell proliferation and migration in vitro [68].

*Selaginella tamariscina* (Beauv.) Spring, which was first recorded in ‘Shen Nong Ben Cao Jing’ (a classical traditional Chinese medicine book) approximately 1700 years ago, and amentoflavone was found to be the main component of the total flavonoids [54]. Chen and his colleagues investigated whether amentoflavone induced anti-angiogenic and anti-metastatic effects through suppression of NF-κB activation, which is a family of transcription factors implicated in various aspects of the tumor biology such as cell proliferation, angiogenesis, metastasis and drug resistance in breast cancer [89]. Obtained results indicated that amentoflavone reduce NF-κB activation, expression and secretion of angiogenesis- and metastasis-related proteins, and cell invasion, so the conclusion is inhibition of NF-κB activation decreases expression and secretion of angiogenesis- and metastasis-related proteins [90]. The formation of blood vessels plays a vital role in the growth of vascular dependent tumor, especially for breast cancer, and angiogenesis plays an important role in its invasion and metastasis. Glabridin, is a polyphenolic flavonoid and is a main constituent in the hydrophobic fraction of licorice extract [91]. Mu and his team studied the underlying molecular mechanisms, and results showed that glabridin attenuated the angiogenic ability via inhibition of microRNA-520a (miR-520a)-mediated NF-κB/IL-6/STAT-3 signalling, the VEGF secretion, and the angiogenesis in MDA-MB-231 breast cancer cell line [55].

Tasquinimod is a small-molecule immunotherapy with demonstrated effects on the tumor microenvironment involving immunomodulation, anti-angiogenesis and inhibition of metastasis [92]. A target molecule of tasquinimod is the inflammatory protein S100A9 which has been shown to affect the accumulation and function of suppressive myeloid cell subsets in tumors. The MC38-C215 colon carcinoma tumors were studied and datas showed that tasquinimod affected tumor infiltrating myeloid cells, leading to a change in phenotype from pro-angiogenic which consistent with the effects of tumor vascularization and metastasis [92]. These results giving further insights to the anti-tumor mechanism of natural polyphenols from a perspective of anti-angiogenesis in breast cancer metastases.

### 2.3. NF-κB

NF-κB is a transcription factor involved in multicellular biological responses [93]. NF-κB signaling has been reported to be intimately involved in bone and liver metastasis [94]. NF-κB exists as an inactive complex with the inhibitory protein I-κB in the cytoplasm in normal resting cells [95], Upon activation, it enters the nucleus where it regulates the expression of diverse genes encoding cytokines, cell adhesion molecules, growth factors, and apoptotic-related proteins [96]. Combined dietary grape natural polyphenols up-regulated FOXO1 and NFKBIA (I-κBα), thus activating apoptosis and potentially inhibiting NF-κB activity [60]. Curcumin suppressed I-κB phosphorylation in MDA-MB-231 breast cancer cells and diminished NF-κB translocation to the nucleus as monitored by the p65 unit of NF-κB. The results also showed that a significant decrease of AP-1 binding upon treatment with curcumin, suggesting an association between NF-κB and the c-Jun/AP-1 pathway [50]. Continuing studies illustrated that curcumin reduced the metastasis of MDA-MB-231 cells by inhibiting NF-κB signaling pathway activation through indication of miR181b expression, miR181b down-regulates the pro-inflammatory cytokine CXCL1, causing subsequent loss of metastatic potential and disrupting the feedback loop between CXCL1/2 and NF-κB [49]. Butein, another natural polyphenol derived from the stembark of cashews and the heartwood of *Dalbergia odorifera*, down-regulates the expression of CXCR4 (a Gi protein-coupled receptor for the ligand CXCL12) in HER2-overexpressing breast cancer cells via transcriptional regulation as indicated by inhibition of NF-κB activation and down-regulation of mRNA expression [45].

### 2.4. EMT

EMT is a key step, that, through loss of intercellular contacts of epithelium-derived cancer cells, up-regulates components of the contractile cytoskeleton, generating a mesenchymal phenotype with highly invasive characteristics that emigrate the primary cancer [97]. Steroid hormones such as estrogen play crucial roles in breast cancer progression. Most responses are mediated through estrogen receptor alpha (ERα) and ERβ [98]. The presence of ERα is considered to be a good prognostic factor and correlates with a higher degree of cancer differentiation [98]. MMP-9 has been previously reported to promote the metastasis of cancer cells. Recent research has shown that high levels of MMP-9 were negatively correlated with ER [99]. EGCG treatment activated Forkhead box O transcription factor, FOXO3a, a major transcriptional regulator of ERα, inhibits the invasive phenotype through activation of ERα signaling in breast cancer cells. The fact that EGCG represses EMT [31], a critical feature of embryogenesis, has been recognized for several decades. Cancer cells lose expression of proteins that promote cell-cell contact such as γ-catenin and E-cadherin and acquire mesenchymal markers such as the zinc-finger transcription factor Snail, fibronectin, N-cadherin, and vimentin during EMT [100]. Curcumin is an antioxidant that exerts antiproliferative and apoptotic effects and has anti-invasive and anti-metastatic properties. The effect of curcumin (30 µM for 48 h) was evaluated on the expression of EMT-related genes. The results showed that curcumin decreased E-cadherin, N-cadherin, β-catenin, Slug, AXL, Twist1, vimentin and fibronectin protein expression, and all of these changes induced a decrease in migratory and invasive capabilities in a breast cancer cell line [51,101]. Health benefits of xanthohumol, a natural extract sourced from hops and beer [102], suppressed markers of EMT and of cell mobility such as paxillin, MCL2 and S100A4, and also attenuated cancer cell-mediated defects at the lymphendothelial barrier, inhibiting EMT-like effects [46]. The Korean annual weed, *Artemisia annua* L., has been used as a folk medicine for treatment of various diseases. Ko and his team investigated anti-metastatic effects of natural polyphenols from Korean *A. annua* L. (pKAL) on the highly metastatic MDA-MB-231 breast cancer cell line, focusing on cancer cell adhesion to the endothelial cell and epithelial-mesenchymal transition (EMT). These results suggest that pKAL exhibits anti-invasive effects through suppression of MMP-2 and MMP-9, two key molecules in proteolytic digestion of ECM. This is consistent with previous reports using quercetin or kaempferol [73,103].

### 2.5. mTOR

mTOR, a central regulator of cell growth, proliferation, differentiation and metastasis, has been intensely studied for over a decade. Recent data has shown that the phosphoinositide 3-kinase (PI3-K)/Akt/mTOR pathway, and mTOR in particular, plays a critical role in the regulation of cancer metastasis [104]. Quercetin, one of the natural polyphenols, in combination with resveratrol and catechin (RQC), has been reported to inhibit the PI3K/Akt/mTOR signaling pathway and breast cancer progression in vitro and in vivo. The results showed that RQC reduces mTOR pathway activation and induces apoptosis via inhibition of Akt and activation of AMPK in breast cancer [59]. Of the RQC natural polyphenols, quercetin is the most effective inhibitor of the PI3K enzyme, with an IC_50_ ≈ 3.8 μM [105], compared with resveratrol that has an IC_50_ ≈ 25 μM [106]. Quercetin at 15 μM in vitro and 15 mg/kg in vivo, inhibits Akt/mTOR signalling, induces cell cycle arrest, and inhibits breast cancer growth and metastasis [36].

### 2.6. Others

Lee-Chang and his team found that cancer metastasis requires an additional player, a unique subset of TGFβ-producing regulatory B cells designated cancer-evoked regulatory B cells (tBregs) [46]. RSV inhibits the generation and function of tBregs by inactivating Stat3 phosphorylation and acetylation [43]. Enterolactone, an active polyphenol metabolite of lignan, inhibited migration and invasion of breast cancer cells via the inhibition of phosphorylation in the FAK/paxillin signaling pathway, which is associated with cell adhesion to the extracellular matrix or to surroundings [57]. Previous research has shown that biotransformation of blueberry juice by *Serratia vaccinii* increases its polyphenolic content and reduces lung metastasis of mammary carcinoma through influencing cellular signaling cascades of breast cancer stem cells, controlling PI3K/AKT, MAPK/ERK, and STAT3 pathways in mammary cancer stem cell inflammatory signaling [72].

## 3. The Molecular Mechanism of Natural Polyphenols on Breast Cancer Metastasis In Vivo

Human cancer xenografts constitute the major percentage of cancer biology models used for cancer drug discovery. These models overcome the disadvantages of in vitro models by containing a microenvironment in which the cancer cells can grow. Examination of molecular mechanisms in animal models treated with natural polyphenols has offered some promising results in terms of treating metastases (Table 1).

### 3.1. Natural Polyphenols Monomers

The administration of resveratrol (20 or 50 mg/mouse) to female BALB/c mice with 4T1-cancer reduced breast cancer growth efficiently inhibited lung metastasis. The mechanism of this process occurred through resveratrol-mediated inactivation of Stat3, which prevented the generation and function of tBregs, including expression of TGF-β [43]. Similarly, in female BALB/c mice that received inoculums of 4T1 cells, the macroscopic appearance of the lungs from untreated and treated mice clearly showed that treatment with resveratrol reduced the number of 4T1 colonies in the lungs. In addition, plasma MMP-9 activity was decreased in response to treatment with resveratrol in mice [40]. However, in another model of mouse breast cancer, hairless SCID and athymic nude female mice bearing MDA-MB-231 and metastatic variant of MDA-MB-435 breast cancers were orally gavaged with 0.5, 5, or 50 mg/kg body weight resveratrol for 35 days. The necropsy reports showed that resveratrol at low concentrations can promote mammary cancer growth and metastasis to lungs, livers, kidneys, and bones, primarily due to a significant induction of Rac activity and a trend in increased expression of the Rac downstream effector PAK1, as well as other cancer promoting molecules. In contrast to other studies, these findings strongly illuminate the importance of delineating resveratrol’s concentration-dependent effects [44]. Therefore, the data reported on administration of resveratrol in animal models of breast cancer metastasis are contradictory. Exposure to 20 mg/kg picetannol significantly reduced the number and volume of pulmonary cancer nodules and expression of MMP-9 in both lung and cancer in nude mice [28]. For this study, BJMC3879 cells were inoculated subcutaneously in female BALB/c mice, which were then given vaticanol C (Vat-C, a novel resveratrol tetramer) at either 100-ppm or 200-ppm in their diet for 8 weeks. The results demonstrated that the multiplicities of lymphatic and pulmonary metastasis were significantly lower in the 200-ppm Vat-C group, as were overall metastasis to any organ [56].

Curcumin has been widely used in Southeast Asian countries as spice or traditional medicine [107]. It has been previously reported that curcumin prevents the formation of hematogenous breast cancer metastases in immunodeficient mice in a highly significant manner. MDA-MB-231 cells were injected into the heart of mice that were then fed diets supplemented with 1% curcumin. The results showed that curcumin administration significantly prevented lung metastases. To explore the mechanism underlying the influence of curcumin on the expression of a series of miRNAs in metastatic breast cancer cells, MDA-MB-231^miR181bt+^ or MDA-MB-231^MOCK^ cells were implanted in the left cardiac ventricle of CD-1 Foxn1^nu^ female mice. The data demonstrated that over-expression of miR181b inhibits metastasis formation, particularly in the lung [49]. However, owing to poor solubility [108] and extensive fast rate of metabolism [109], the therapeutic efficacy of curcumin was limited. Therefore, curcumin loaded biodegradable self-assembled polymeric micelles (Cur-M) were prepared and investigated for their treatment effect on the subcutaneous 4T1 breast cancer model with Cur-M treatment (30 mg/kg) for ten days. These findings indicated Cur-M not only blocked implanted cancer growth but also impaired cancer metastasis [58]. Exposure to 80 mg/kg dendrosomal curcumin significantly decreased metastasis formation in the lung, the sternum and the liver of BALB/c mice injected subcutaneously with 4T1 cells. The results also reported suppression of NF-κB expression via down-regulation of VEGF, COX-2, and MMP-9 expression in the breast cancer as well as in brain, lung, liver and spleen tissues [48].

Likewise, mice subcutaneously injected with MCF-7 cells and exposed to oleuropein at 125 mg/kg demonstrated for the first time that oleuropein prevented both peripulmonary and parenchymal lung metastases [53]. Otherwise, female SCID mice with MDA-MB-231 cells were exposed to 15 or 45 mg/kg quercetin. The results show that quercetin treatment decreased metastasis [36].

### 3.2. Combined Natural Polyphenols and Other Antimetastatic Drugs

The effects of combining natural polyphenols with conventional drugs in animal models are necessary to evaluate their pharmacodynamic actions, and lay the foundation for preclinical study. Tea catechins in combination with anticancer drugs are being evaluated as a new cancer treatment strategy [110]. Combined administration of EGCG and curcumin (25 mg/kg & 200 mg/kg, respectively) in athymic female mice implanted with ERα-breast cancer cells showed reduced tumor volume with decreased VEGFR-1 expression [111]. Administration of EGCG and taxol in BALB/c mice injected with 4T1 mouse breast cancer cells dramatically decreased cancer growth and numbers of the lung metastases, however, there were no significant effects when mice were exposed to EGCG or taxol alone [112]. Another line of evidence for synergism between paclitaxel and natural polyphenols was presented by Kang and colleagues, who discovered that the combination of paclitaxel and curcumin decreased cancer cell proliferation, increased apoptosis, and decreased expression of MMP-9 in a breast cancer murine model using MDA-MB-231 cells. This study demonstrated the combined effects of the paclitaxel and natural polyphenols, illustrating that this two compounds combine to inhibiting cancer metastasis [113].

MDA-MB-231 cells were injected into hairless severe combined immunodeficiency (SCID) female mice to produce orthotopic primary cancers, and mice were orally gavaged with a combination of 5 mg/kg RQC and 200 mg/kg gefitinib, or treatments were given separately. The results demonstrated combined RQC and gefitinib was more efficient than either treatment alone at inhibiting mammary cancer growth and metastasis via inhibition of Akt signaling and mTOR. Activation of AMPK occurred even in the presence of gefitinib [59]. Similarly, the administration of RQC (5 mg/kg) to female nude mice with MDA-MB-435 cells reduced metastasis especially to liver and bone [60]. The results demonstrated that natural polyphenols generally increase conventional drug efficacy, making them attractive candidates for adjuvant therapy against metastatic cancer.

### 3.3. Natural Polyphenols Extracts

*Camellia sinensis* (0.6 g/kg) was effective in reducing tumor weight by 34.8% in female BALB/c mice compared to the control group that received water treatment (100%). In addition, *C. sinensis* treatment significantly decreased lung and liver metastasis by 54.5% and 72.6%, respectively [61]. MDA-MB-435/HAL cells were injected into female athymic nude mice, and various experimental designs were used to assess the anti-metastatic effect of the soy isoflavone genistein through postsurgical dietary intervention. The results demonstrated that the growth ability of previously seeded and potentially metastatic cells could be affected by dietary intervention following surgical resection and then the application of a diet enriched in genistein. This anti-metastatic action illustrates the potential of genistein as a postsurgical adjuvant therapy for ER–negative breast cancer, which has been supported by additional studies [39]. However, dietary enrichment in soy isoflavones has also been associated with increases in Ki-67 protein expression and metastatic lung cancer formation [63]. These studies illuminate the need for further delineating polyphenolic effects, especially in breast cancer, before these compounds can be tested in the clinic. Mice feda diet supplemented with *Murraya koenigii* (50 and 200 mg/kg) for 30 days were subsequently inoculated with different numbers of 4T1 cells in low and high-risk cancer groups. *Murraya koenigii* treatment continued until day 21 post-inoculation, and the reported data showed that *M. koenigii* reduced lung metastasis, and diminished the level of nitric oxide and inflammation-related cytokines and genes, including NF-κB, iCAM, c-MYC and iNOS [70]. The anti-metastatic effects of peach polyphenolics were investigated using a xenograft model with MDA-MB-435 breast cancer cells in vivo. Mice received peach phenolics (0.2- to 1.6-mg *chlorogenic acid equivalent* (CAE)/day) by oral gavage. The results showed that lung metastases were inhibited through inhibition of MMPs gene expression [66]. In addition, the specific phenolic compounds derived from peaches and plums have been demonstrated to suppress tumor growth and metastasis in xenograft models [114]. Using a xenograft model in which MDA-MB-435 was cultured with *peach phenolics* (1.6-mg CAE/day), which equates to the equivalent human intake of two to three peaches every day, peach phenolics were able to inhibit gene expression of MMP-2, MMP-13 and MMP-3 [66]. Artichokes decreased the proteolytic activity of MMP-2, which is involved in degrading the components of the ECM [67].

**Figure 4:**
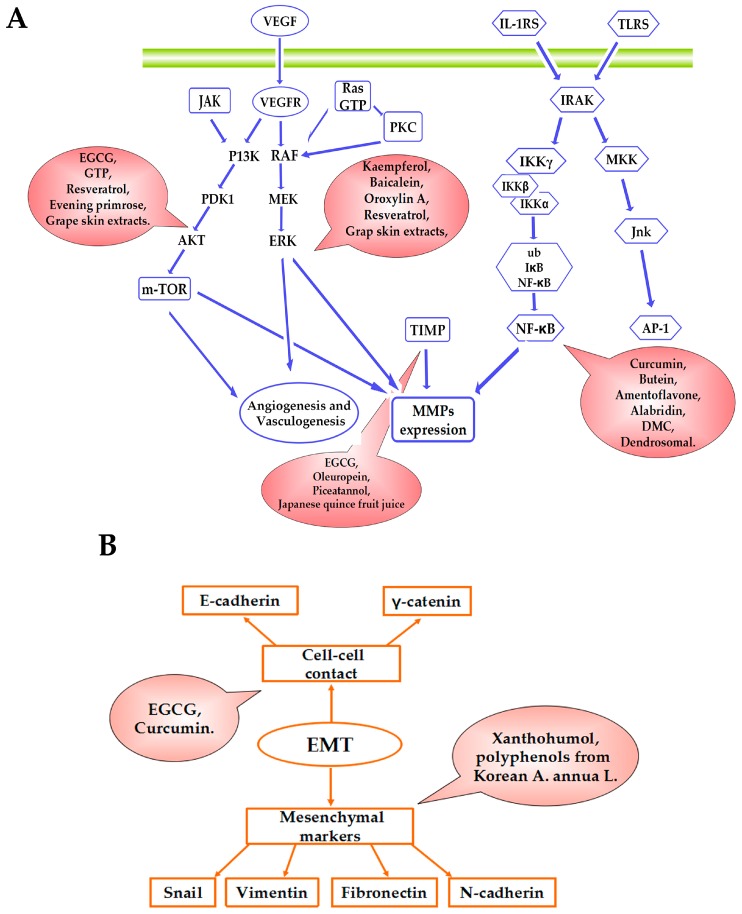
Model for the molecular metchanisms of natural polyphenols effects on metastasis. (**A**) General schematic representation of signalling pathway that regulate MMP-2 and MMP-9 expression, VEGF gene and NF-κB; (**B**) The proteins that promote cell-cell contact and mesenchymal markers during EMT.

In summary, an increasing amount of research has been dedicated to identifying potential mechanisms underlying the suppression of breast cancer metastasis by natural polyphenols. Proposed molecular mechanisms include down-regulation of MMPs expression, modulation of EMT regulators, interfering with VEGF signaling, inhibition of NF-κB and mTOR expression, and other possible mechanisms (as shown in Figure 4). In addition, many researchers have described the biological phenomenon of metastasis and its mechanism of action. The complexity of natural polyphenol extract is well known, so further exploring the effects of natural polyphenol metabolism in the context of cancer is the foundation of continued molecular mechanism studies.

## 4. Metabolomics

Metabolomics is a powerful technology in pharmaceutical and clinical research that describes the similarities and differences between biological samples by profiling and comparing the metabolite panel in an organism, resulting in the development of personalized methods for patient monitoring, treatment response evaluation, and disease diagnosis [115]. Natural polyphenols are transformed into a variety of diverse metabolites in the body [116], some of which are still unidentified [117]. Metabolomics provides an effective method to simultaneously detect hundreds of natural polyphenols or their metabolites in a global way. Metabolomics results have been utilized for analyzing the molecular mechanism of diseases, such as malignant tumors [118], and to classify food and plant species [119] in recent decades. High-throughput analytical methods such as NMR spectroscopy, liquid chromatography-quadrupole time-of-flight (LC-QTOF) [120] or MS allow simultaneous analysis of hundreds of metabolites forming the metabolome from urine or plasma [121]. Table 2 showes the modifications of endogenous metabolites derived from natural polyphenols intake.

### 4.1. Natural Polyphenols

Previous studies have used various metabolomic approaches to explore the changes in metabolic profiles induced by hyperlipidemic diets. A relatively high urine excretion of deoxycytidine nucleosides has been closely linked to a higher risk for immune deficiency syndrome and cancer [127]. An improvement of deoxycytidine excretion in urine achieved with high-fat diets might result from an increase in DNA breakdown or a higher production of deoxycytidine by enteric bacteria [128]. Peroxisomal or chronic liver dysfunction disorders [129] result in high urinary content of pipecolinic acid [130]. Similarly, the origin of the variations in the urinary excretion of dihydroxyquinoline and nicotinic acid observed following high-fat diets. After catechin supplementation, the excretion of the compounds mentioned above returned to the level observed in rats fed with the low-fat diet [120]. Inspection of the principal component (PC) loadings followed by subsequent examinition of the corresponding urinary ^1^H-NMR spectra enabled identification of the endogenous metabolites induced from exposure to EC, and the levels were perturbed. The spectral descriptors most affected by EC dosing were found to correspond to a reduction in the urinary concentrations of 2-oxoglutarate, citrate, dimethylamine, creatinine, and taurine [122]. This metabolite could be impacting the effects of dietary natural polyphenols on liver and kidney function and could reflect a shift in energy metabolism from carbohydrate metabolism to lipid or amino acid metabolism [131].

### 4.2. Polyphenolic Extracts

Natural polyphenols constitute 10%–12% of the dry weight of green and black tea leaves and black and green tea consumption have different effects on human metabolism. The consumption of green tea indicated that green tea polyphenols may be affecting oxidative energy metabolism or biosynthetic pathways. Black tea increased levels of ketone body β-hydroxybutyrate in urinary excretion, suggesting impacts on fatty acid oxidation and liver ketogenesis [123]. It has been shown that green tea can protect against the advancement of a series of chronic diseases [132]. In particular, studies have focused a great deal on the prevention of cancer [133] and cancer metastasis [134]. The pharmacokinetics and bioavailability of green tea extract (GTE) catechins following a single bolus dose intake may induce significant acute impacts on multiple endogenous metabolites. This differs from the metabolite changes observed after one-week of continued supplementation. Long-term GTE supplementation is necessary to achieve regulation of various enzymes and proteins at certain sites in the body, leading to systemic shifts in levels of the endogenous metabolites, while acute metabolic effects may suffer from different mechanisms, while acute metabolic events may be occurring by distinct mechanisms. The acute effects on the phosphatidylcholines and cholesterylesters may point to changes in lipid metabolism. Other changes, for instance in sertonine, sphingosines and salicylic acid may be correlated with vascular function [124].

Pu-erh tea, a fermented tea including large quantities of polyphenolic constituents [135], has been shown to exert preventive and anti-metastatic effects on oral cancer and on buccal mucosa cancer [136]. Valuable and complementary information on Pu-erh tea degradation was provided by the combined UPLC-QTOFMS and GC-TOFMS based metabolomics technology. One of the tea metabolites, caffeines, was positively correlated with its biodegradated metabolites paraxanthine, paraxanthine, Theophylline was positively corrected with valine, tyrosine, ornithine, and 2-methylguanosine, whereas theophylline was positively corrected with 2-methylguanosine but negatively corrected with aminomalonic acid and urea [125]. Understanding the metabolic characteristics of Pu-erh tea may accelerate the development of its pharmacological properties in preclinical studies.

Using a metabolomics technology to explore the metabolic effect of polyphenol-rich red wine and grape juice (MIX) consumption in humans, gas chromatographymass spectrometry (GC-MS) was used for focused profiling of urinary phenolic acids, while ^1^H-NMR spectroscopy was used for global metabolite analysis. After a 4-week intake of the MIX, hippuric acid, an index of gut microbiota-mediated degradation of dietary natural polyphenols, increased significantly in urine samples. Noticeablely, 3-methoxy-4-hydroxymandelic acid, phenylacetylglutamine and 4-hydroxymandelic acid production results from the modulation of endogenous biological pathways, not metabolites of dietary natural polyphenols [126]. Overall, MIX polyphenols seem to have a slight effect on endogenous metabolism, most obviously in several amino acid derivatives containing bacterial metabolites of tryptophan and tyrosine. These results demonstrate that short-term intake of MIX or their metabolites leads to altered microbial amino acid metabolism and microbial protein fermentation [137].

## 5. Conclusions and Future Perspectives

Natural polyphenols plays a significant role in the prevention of breast cancer. Many studies have shown the capabilities of natural polyphenols to suppress breast cancer cell migration and invasion and to inhibit metastasis formation both in vitro and in vivo. More and more research is devoted to studying the potential mechanisms of breast cancer metastasis from the cascades of breast cancer metastasis. These molecular and cellular mechanisms include down-regulation of MMPs expression, interference with the VEGF signaling, modulation of EMT regulators, inhibition of NF-κB and mTOR expression, and other creditabe mechanisms.

Breast cancer distant metastases rank first among causes of cancer-related deaths, and the incidence of brain metastases continues to increase with current estimates in the U.S. [138]. Unfortunately, clinical trials aimed at treating brain metastases using traditional breast cancer drugs have produced little success [139,140], likely due in part to the blood-brain barrier (BBB) and the blood-cancer barrier (BTB), which establish a protective environment for metastatic growth, preventing the absorption of chemotherapy drugs. Thus, more work should be done to explore the effect of natural polyphenols on breast cancer brain metastasis, looking at the advantages of molecular diversity, which can increase CNS penetration. Of course, natural polyphenols are unlikely to be anticancer agents when used individually; however, their use as an adjuvant to chemtherapy drugs may help impede the progression of breast cancer metastasis.

## Figures and Tables

**Table 1:** Mechanisms of natural polyphenols interfering in breast cancer metastasis.

Natural Polyphenols	Cancer Type or Animal Type	Effective Concentrations or Doses	Result	Ref.
(−)-Epigallocatechin Gallate (EGCG) 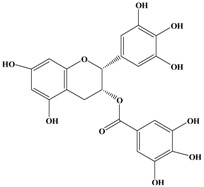	MCF-7	In vitro: 30 μM	Suppressing the Heregulin β1-stimulated activation of epidermal growth factor receptor-related protein B2 (ErbB2)/ErbB3/protein kinase B.	[30]
	Mouse mammary cancer virus-Her-2/neu cell line NF639	In vitro: 60 μg/mL	Up-regulation of the epithelial genes *E-cadherin*, *γ-catenin*, *MTA3*, and *estrogen receptor α (ERα)*. Down-regulation of proinvasive *snail* gene. Activation of FOXO3a.	[31]
	Inflammatory Breast Cancer lines: SUM-149 and SUM-190	In vitro: 5–160 μg/mL	Elevation of the levels of cleaved Caspase-3 and PARP.	[32]
	MCF-7 MDA-MB-231	In vitro: 20 μM	Reducing EZH2 and class I HDAC protein levels, inducing TIMP-3 levels,suppressing invasiveness and activity of MMP-2 and MMP-9.	[26]
(−)-Epigallocatechin (EGC) 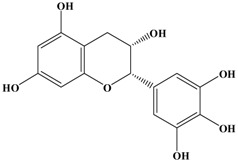	MDA-MB-231 human breast cancer cell line	In vitro: 50, 80 μg/mL	Inhibition of MMP-9 expression and AKT signaling pathway by inhibiting both at the RNA and protein level.	[33]
	MCF-7	In vitro: 30 μM	Disruption of the Heregulin β1-stimulated activation of ErbB2/ErbB3/Akt.	[30]
(−)-Epicatechin 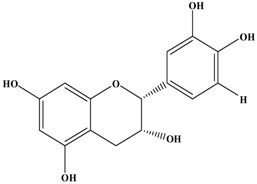	Murine mammacarcinoma cell line 4T1	In vitro: 10 μM	Inhibiting cell shedding and invasion by their anti-oxidative capacity and down-regulation of MMP-9 expression.	[34]
Kaempferol 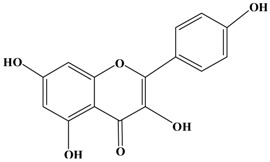	MDA-MB-231 human breast carcinoma cells	In vitro: 10, 20, 40 μM	Blocking the PKCδ/MAPK/AP-1 cascade and subsequentiy suppressing MMP-9 expression.	[35]
Quercetin 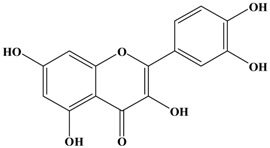	Metastatic MDA-MB-231 MDA-MB-435 Female severe combined immunodeficiency (SCID) mice	In vitro: 15 μM In vivo: 15 mg/kg 3*X* per week i.p.	Induction of mTOR activities through Akt inhibition and AMPK activation.	[36]
Oroxylin A 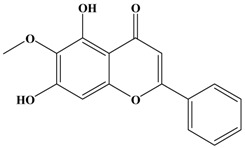	MDA-MB-435	In vitro: 1, 10 and 100 μM	Inhibiting the expressions of MMP-2, MMP-9 and ERK1/2	[37]
Baicalein 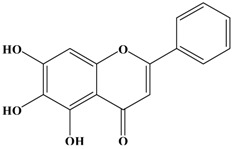	MDA-MB-231	In vitro: 2, 10, 50 μM	Down-regulating the expression of MMP-2/9 involved MAPK signaling pathway.	[38]
Genistein 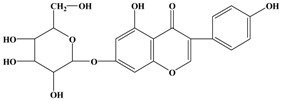	MDA-MB-435/HAL	In vivo: 750 μg/g	Reducing the percent metastatic burden in the lungs, and affect the outgrowth of seeded tumor cells by dietary intervention following cancer surgery.	[39]
Resveratrol 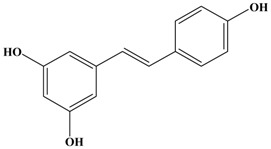	4T1 Female BALB/c mice	In vitro: 0–30 μM In vivo: 100 mg/kg/day 200 mg/kg/day	Decreasing the activity and expression of MMP-9.	[40]
	MCF-7	In vitro: 2, 5 and 10 μM	Inhibition of HRG-β1-mediated MMP-9 expression via down-regulation of the MAPK/ERK signaling pathway.	[41]
	MDA-MB-435	In vitro: 10 and 20 μM	Suppressing insulin-like growth factor (IGF)-1-mediated cell migration and invasion and MMP-2 expression via inhibition of the PI3K/Akt signaling pathway.	[42]
	Female BALB/c mice (4T1)	In vivo: 50 mg/mouse/2day i.p.	Inactivating Stat3, preventing the generation and function of tBregs, including expression of TGF-β.	[43]
	Immunocompromised mice Green fluorescent protein (GFP) tagged-MDA-MB-231 (ERα(-), ERβ(+)) or a metastatic variant of GFP-MDA-MB-435 (ER (-)) cells	In vivo: 0.5, 5 and 50 mg/mouse 5 days/week i.g.	Increased expression of the Rac downstream effector PAK1, JNK and Akt.	[44]
Piceatannol 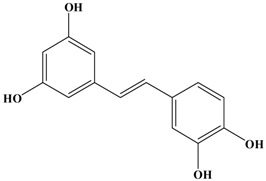	Female BALB/c mice (4T1)	In vivo: 10 and 20 mg/kg/day i.g. In vitro: 30 μM	Reducing the expression of MMP-9 in both cancer and lung tissues and increasing apoptotic cells and expression of both Bax and cleaved caspase-3 but reducing Bcl-2 expression in cancer tissues.	[28]
Butein 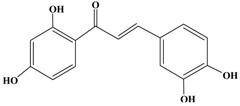	HER2-over-expressing breast cancer SKBr3 cells	In vitro: 10,25,50 μM	Inhibition of CXCR4 expression correlated with the suppression of CXCL12- induced migration and invasion, and inhibiting of NF-κB activation.	[45]
Xanthohumol 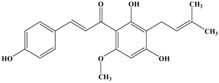	MCF-7 MDA-MB-231	In vitro: MCF-7: 5 μmol/L MDA-MB-231: 50 μmol/L	Inhibiting the activity of CYP, SELE and NF-κB, affecting ICAM-1 expression and adherence to LECs, suppressing paxillin, MCL2 and S100A4.	[46]
Curcumin 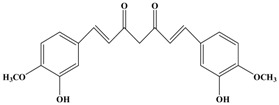	MCF-7	In vitro: 30 μM	Suppression of the PKCα, MAPK and NF-κB/AP-1 pathway and TPA-induced MMP-9 expression	[47]
	4T1 BALB/c mice	In vitro: 5–20 μM In vivo: 40 mg/kg 80 mg/kg	Suppression of NF-κB expression by down-regulation of VEGF, COX-2, and MMP-9 expressions.	[48]
	MDA-MB-231 MDA-MB-231^MOCK^ injecting into the left cardiac ventricle CD-1 Foxn1nu female mice.	In vitro: 25 μM	Up-regulation of miR181b and down-regulation of CXCL1 and 2.	[49]
	MDA-MB-231 MDA-MB-231^MOCK^ injecting into the left cardiac ventricle CD-1 Foxn1nu female mice.	In vitro: 25 μM In vivo: feding with standard diet containing 1% curcumin	Reducing the expression of MMPs via down-regulation of NF-κB and AP-1 activity and transcriptional.	[50]
	MCF-10F; MDA-Mb-231; Tumor 2	In vitro: 30 µM for 48 h	Decreasing E-cadherin, N-cadherin, β-catenin, Slug, AXL, Twist1, Vimentin and Fibronectin protein expression involved in EMT.	[51]
Demethoxycurcumin 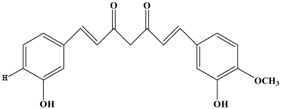	MDA-MB-231	In vitro: 1, 7.5, 15 μM	Inhibiting the DNA binding activity of NF-κB, decreasing the levels of ECM degradation-associated proteins including MMP-9, MT1-MMP, uPA , uPAR, ICAM-1 and CXCR4, up-regulating the level of PAI-1.	[52]
Oleuropein 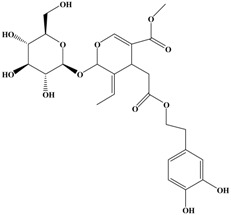	MDA-cell line	In vitro: 200 μg/mL	Increasing the TIMPs, and then suppressing the MMPs expressions	[27]
	Ovariectomised nude mice MCF-7	In vivo: 125 mg/kg diet	Possessing a potent in vivo anti-cancer activity inhibiting both the MCF-7 cells xenograft growth and their invasiveness into the lung.	[53]
Amentoflavone 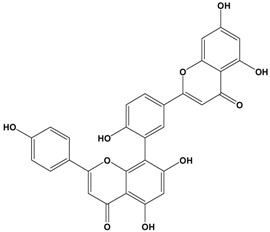	MCF-7	In vivo: 50 and 100 μM in 0.1% DMSO	Inhibiting NF-κB activation decreases expression and secretion of angiogenesis- and metastasis-related proteins. Amentoflavone may induce anti-angiogenic and anti-metastatic effects through suppression of NF-κB activation.	[54]
Glabridin 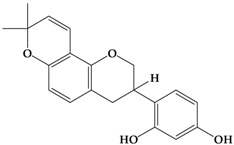	MDA-Mb-231	In vitro: 8 μM for 24, 48, 72 h	Attenuating the angiogenic ability by the microRNA-520a (miR-520a)-mediated inhibition of the NF-κB/IL-6/STAT-3 signal pathway.	[55]
A resveratrol tetramer	BJMC3879 Female BALB/c mice	In vitro: 8 μM In vivo: 22.9 mg/kg/day 44.7 mg/kg/day i.p.	Modulating the transcription level of mutant *p53*. Suppressing metastasis to both lymph nodes and lungs.	[56]
Enterolactone, an active polyphenol metabolite of Lignan	MDA-MB-231 cells	In vitro: IC_50_ = 261.9 ± 10.5 μM	Down-regulating phosphorylation of the FAK/paxillin pathway, inhibiting migration and invasion of cells.	[57]
Curcumin loaded polymeric micelles	Subcutaneous 4T1 breast cancer model	In vivo: 30 mg/kg/day	Inhibiting cancer growth and spontaneous pulmonary metastasis.	[58]
Grape natural polyphenols: resveratrol,quercetin,catechin,(or combination with gefitinib)	Human metastatic breast cancer cell GFP-MDA-MB-231 (ERα(−), ERβ(+)) Immunocompromised mice	In vitro: 5 μM In vivo: 5 mg/kg/day respectively i.g. or 5 mg/kg/2days and 200 mg/kg/2days i.g.	Reducing Akt activity, inducing the activation of AMPK and inhibiting mTOR signaling pathway.	[59]
	ER(−) GFP-MDA-MB-435 Female athymic nu/nu mice	In vitro: 0.5 or 5 μM In vivo: 5 mg/kg/day together i.g	Up-regulating FOXO1 and NFKBIA (IκBα), activating apoptosis and inhibiting NF-κB activity, reducing metastasis.	[60]
Mixed natural polyphenols resveratrol, baicalein, epicatechin, epigallocatechin polyphenon 60	4T1 multicellular cancer spheroids	In vitro: 100 μM	Suppressing invasion by down-regulation of MMP-9 expression and their anti-oxidative capacity.	[34]
Natural polyphenols extract from Green tea	MCF-7 MDA-MB-231	In vitro: 10 μg/mL	Reducing EZH2 and class I HDAC protein levels, inducing TIMP-3 mRNA and protein levels, suppressing invasiveness and activity of MMP-2 and MMP-9.	[26]
	Mouse mammary carcinoma 4T1 cells Female BALB/c mice	In vitro: 0.06–0.125 mg/mL In vivo: 0.6 g/kg/day i.g.	Increasing the expression of Bax-to-Bcl-2 ratio, activating caspase-8 and caspase-3, decreasing lung and liver metastasis, protecting the bone from breast cancer-induced bone destruction.	[61]
	Mouse mammary carcinoma 4T1 cells Female BALB/c mice	In vivo: 0.2% and 0.5% *w*/*v* in drinking water and was started 7 days before cancer cells inoculation	Decreasing the protein expression of Bcl-2 concomitantly increase in Bax, cytochrome c release, Apaf-1, and cleavage of caspase 3 and PARP proteins,inhibiting lungs metastasis.	[62]
Natural polyphenols extract from Green tea	MDA-MB-231 human breast cancer cell line	In vitro: 40, 60 μg/mL	Inhibition of MMP-9 expression and AKT signaling pathway by inhibiting both at the RNA and protein level.	[33]
Natural polyphenols extract from Soy isoflavones	Female BALB/c mice (4T1)	In vivo: 750 mg/kg/day diet genistein equivalent	Increasing Ki-67 protein expression amd stimulating metastatic cancer formation in lungs.	[63]
Natural polyphenols extract from Japanese quince fruit	MDA-MB-231	In vitro: 25, 50, 75, 100 μM catechin equivalents	Decreasing the MMP-9 activity and stimulating the TIMP-1 expression.	[29]
Natural polyphenols extract from *Nelumbo nucifera Gaertn* leaves	MDA-MB-231	In vitro: 0.5~2.0 mg/mL	Blocking vascular-like structure formation, suppressing CTGF expression reducing the MMP2 and VEGF expression, and attenuating PI3K-AKT-ERK activation.	[64]
Natural polyphenols extract from *Leucobryum bowringii* Mitt.	MCF-7	In vitro: 10, 25 and 50 μg/mL	Inhibition of MMP-2 and MMP-9 activities.	[65]
Natural polyphenols extract from Peach phenolics	MDA-MB-435	In vivo: 0.8–1.6 mg chlorogenic acid equivalent /day i.g.	Down-regulating the gene expression of MMPs, and up-regulating hβ2G gene expression in the lungs.	[66]
Natural polyphenols extract from Artichoke	MDA-MB-231	In vitro: 200 μM	Decreasing of proteolytic activity of MMP-2, involved in degrading components of the extracellular matrix.	[67]
Natural polyphenols extract from Grape skin	4T1 cells Balb/c mice implanting 4T1 subcutaneously	In vitro: 0.5 and 1.0 mg/ml in drinking water	Blocking the PI3k/Akt and MAPK pathways.	[68]
Natural polyphenols extract from *Evening primrose*	MDA-MB-231	In vitro: IC_50_ = 58 μM (gallic acid equivalents)	Decreasing the activity of MMP-9 through reducing the expression levels of the following proteins: VEGF, c-Fos, c-Jun.	[69]
Natural polyphenols extract from *Murraya koenigii*	MDA-MB-231 Left mammary fat pad subcutaneously 4T1	In vitro: MDA-MB-231 (IC_50_ = 2.40 ± 0.26) 4T1 (IC_50_ = 1.50 ± 0.90) In vivo: 50 mg/kg 200 mg/kg	Decreasing the level of nitric oxide and inflammation-related cytokines and genes, including iNOS, iCAM, NF-κB and c-MYC and reducing lung metastasis.	[70]
Natural polyphenols extract from Grape seed proanthocyanidins	4T1 cells were implanted subcutaneously in Balb/c mice	In vivo: 0.2% and 0.5%, *w*/*w* in a diet	Increasing the ratio of Bax:Bcl-2 proteins, cytochrome c release, induction of Apaf-1 and activation of caspase 3, inhibiting the metastasis of cancer cells to the lungs.	[71]
Natural polyphenols extract from biotransformation of blueberry juice by *Serratia vaccinii*	murine 4T1 human MCF7 and MDA-MB-231 BALB/c mouse model	In vitro: 100 μM (gallic acid equivalent) In vivo: 2.9 mL/day	Decreasing lung metastasis by controlling PI3K/AKT, MAPK/ERK.	[72]
Natural polyphenols extract from Korean *A. annua* L.	MDA-MB-231	In vitro: 1, 10, 30 ug/mL	Inhibiting the cancer cell adhesion to ECs through suppression of vascular cell adhesion molecule-1 and invasion through suppression of EMT, MMP-2 and MMP-9.	[73]

**Table 2:** Catechin intake resulting in endogenous metabolite modifications.

Intervention	Subjects (Samples)	Analytical-Technique	Modified Endogenous Metabolites	Biological Hypotheses	Ref.
*Animal study*: normolipidemic (5% *w*/*w*) or hyperlipidemic (15 and 25%) diets with or without catechin supplementation (0.2% *w*/*w*).	Male Wistar rats (urine)	LC-QTOF	↑ Pipecolinic acid ↑ Nicotinic acid ↑ Dihydroxyquinoline ↑ Deoxycytidine	Possible inhibition of microbiota growth by catechin. Chronic liver dysfunction or peroxisomal disorders and increase in DNA breakdown.	[120]
*Animal study*: a single dose of 22 mg of epicatechin	220–270 g male Sprague-Dawley (SD) rats (urine)	^1^H-NMR	↓ Taurine ↓ Creatinine ↓ Dimethylamine ↓ 2-Oxoglutarate ↓ Citrate.	Modification in carbohydrate metabolism; Changes in liver and kidney functions.	[122]
*Human study*: Consumption of green tea (6 g/day), black tea (6 g/day) or caffeine (control) for 2 days	17 nonsmoking male (urine and plasma)	^1^H-NMR	↑ Succinate ↑ Oxaloacetate ↑ 2-oxoglutarate	Stimulation of oxidative energy metabolism	[123]
*Human study*: a single dose (acute) of GTE or placebo (PLA) and following 1 day, 7 days, GTE (2 × 559 mg catechins/day, 120 mg caffeine/day), or PLA supplementation	(age 22 ± 5 year, weight 78 ± 10.6 kg) 39 healthy physically active male (plasma)	HPLC-MRM-MS	↑ Caffeine ↑ Taurine ↑ 3,4-Dihydroxyphenyle thylene glycol ↓ Hippurate ↑Salicylate ↑ Fatty acids ↑ Serotonin ↑ Triglycerides ↓ Cholesterylesters and ↑ Sphingosines	Influencing the changes in lipid metabolism and vascular function.	[124]
*Human study*: a dose equivalent to 5 cups of commercially prepared tea.	Range 22–32 years healthy men and women (urine)	UPLC-QTOFMS and GC-TOFMS	↑ ornithine ↑ valine ↑ tyrosine ↑ 2-methylguanosine ↑ 2-aminobutyric acid ↓ urea	Pu-erh tea metabolites	[125]
*Human study*: MIX and the GJX supplements was 800 mg gallic acid equivalents (GAEs) per day for 4 weeks.	(Age: 18–70 years) 33 men and 25 women	^1^H-NMR GC-MS	↑ Nitric oxide ↑ Phenylacetylglutamine ↑ 4-hydroxymandelic acid ↑ Vanillylmandelic acid ↑ Homovanillic acid Urine:↑ Hippuric acid	Promoting vascular endothelial function, indicator of gut microbiota-mediated degradation, benefiting the neurological or cardiovascular health.	[126]
